# Early Induction of Oxidative Stress in Mouse Model of Alzheimer Disease with Reduced Mitochondrial Superoxide Dismutase Activity

**DOI:** 10.1371/journal.pone.0028033

**Published:** 2012-01-19

**Authors:** Hyun-Pil Lee, Neel Pancholi, Luke Esposito, Laura A. Previll, Xinglong Wang, Xiongwei Zhu, Mark A. Smith, Hyoung-gon Lee

**Affiliations:** 1 Department of Pathology, Case Western Reserve University, Cleveland, Ohio, United States of America; 2 Department of Neurology and Neuroscience, Gladstone Institute of Neurological Disease, University of California San Francisco, San Francisco, California, United States of America; Federal University of Rio de Janeiro, Brazil

## Abstract

While oxidative stress has been linked to Alzheimer's disease, the underlying pathophysiological relationship is unclear. To examine this relationship, we induced oxidative stress through the genetic ablation of one copy of mitochondrial antioxidant superoxide dismutase 2 (Sod2) allele in mutant human amyloid precursor protein (hAPP) transgenic mice. The brains of young (5–7 months of age) and old (25–30 months of age) mice with the four genotypes, wild-type (Sod2^+/+^), hemizygous Sod2 (Sod2^+/−^), hAPP/wild-type (Sod2^+/+^), and hAPP/hemizygous (Sod2^+/−^) were examined to assess levels of oxidative stress markers 4-hydroxy-2-nonenal and heme oxygenase-1. Sod2 reduction in young hAPP mice resulted in significantly increased oxidative stress in the pyramidal neurons of the hippocampus. Interestingly, while differences resulting from hAPP expression or Sod2 reduction were not apparent in the neurons in old mice, oxidative stress was increased in astrocytes in old, but not young hAPP mice with either Sod2^+/+^ or Sod2^+/−^. Our study shows the specific changes in oxidative stress and the causal relationship with the pathological progression of these mice. These results suggest that the early neuronal susceptibility to oxidative stress in the hAPP/Sod2^+/−^ mice may contribute to the pathological and behavioral changes seen in this animal model.

## Introduction

Alzheimer's disease (AD) is an age-related neurodegenerative disorder and the increase of oxidative stress has been linked to the progression of the disease. Interestingly, transgenic mouse models of AD also readily display increases in oxidative stress [1]–[3]. Specifically, the indicators of oxidative stress lipid peroxidation and heme oxygenase-1 (HO-1) induction are found to be increased in a transgenic mouse model overexpressing mutant amyloid-β precursor protein (APP) [1]. While the role of amyloid-β (Aβ) in AD development is supported by many studies [4], the long-standing hypothesis that mitochondrial dysfunction and oxidative stress also play fundamental roles in the disease has recently gained experimental traction and renewed interest [5]–[8]. In fact, oxidative damage is now recognized as one of the earliest changes in both familial and sporadic forms of AD [9]–[11].

Superoxide dismutase 2 (Sod2) is an antioxidant enzyme which detoxifies superoxide radicals within the mitochondrial matrix. Mice that have disruptions in the Sod2 gene accumulate nucleic acid oxidative damage in the brain [12] while fertility and lifespan of these hemizygous Sod2+/− and wild type animals are identical [13], [14]. Interestingly, combining the reduction of Sod2 activity with hAPP transgenic mice accelerates several features of AD-like pathology including behavioral changes and development of cerebrovascular amyloidosis [15]. However, specific changes in oxidative stress and the causal relationship with the pathological progression of these mice has not been fully analyzed [15], [16]. In this study, therefore, we assessed the effect of Sod2 reduction in this animal model of AD by measuring the extent of oxidative stress in both young mice at 5–7 months of age, in which amyloid deposition is not yet apparent, as well as in aged mice, at 25–30 months of age. Levels of the lipid peroxidation adduct 4-hydroxy-2-nonenal (HNE) pyrrole and HO-1, which are increased during periods of oxidative stress, were analyzed in the brains of old and young mice. Parallel to the finding in AD where the level of HNE and HO-1 is dramatically increased in the vulnerable neurons [17], [18], reduced Sod2 in mice overexpressing APP results in significantly increased oxidative stress, most notably in younger mice. Thus, reduced Sod2 activity together with mutant hAPP overexpression increases oxidative stress and may facilitate the disease progression and increase the severity of AD-like pathology found in this APP transgenic mouse model.

## Materials and Methods

### Transgenic mice

All the transgenic mouse strains used in this study (*Sod2* ^+/+^, *Sod2* ^+/−^, hAPP/*Sod2* ^+/+^, and hAPP/*Sod2* ^+/−^) has been described in the previous study [19]. All protocols involving the use of mice were approved by the Institutional Animal Care and Use Committee of the University of California, San Francisco.

### Immunohistochemical analyses

For immunohistochemical analysis, 8 µm paraffin-embedded sections from the brains of 26 young mice (5–7 months of age; n = 6−7 per group) and 20 old mice (25–30 months of age; n = 4−6 per group) were prepared and blinded for the analysis. All experiments were carried out by an experimenter blinded to genotype. Slides of all mice were immunostained simultaneously under identical conditions, using the peroxidase-anti-peroxidase method. Initially, slides were deparaffinized in xylene and then transferred through a graded ethanol series followed by incubation in Tris-buffered saline (50 mM Tris, 150 mM NaCl, pH = 7.6, TBS) to rehydrate the tissue. Subsequently, the tissues were incubated overnight with the primary antibodies directed against HNE-pyrrole [17], HO-1 [18], GFAP (Zymed Laboratories). The following day, species-specific secondary antibodies and PAP complex were applied, then the slides were rinsed in Tris buffer and developed for 5 minutes with the chromagen 3′-3′-diaminobenzadine (Dako) as previously described [20]. Finally the slides were dehydrated, mounted with a coverslip and analyzed.

For double immunohistochemistry, the brain sections were incubated overnight at 4°C with anti-HO-1 rabbit antibody or anti- HNE pyrrole rabbit antibody in addition to anti-GFAP mouse monoclonal antibody. Alexa Fluor 488- and 568-coupled secondary antibodies (Invitrogen, Carlsbad, CA) were used for detection. Images were acquired through an AxioCam camera on an Axiovert 200 M microscope (Zeiss, Thornwood, NY). Images were then analyzed with the Axiovision software (Zeiss).

### Image analysis

Quantitative analysis of the neuronal accumulations of the oxidative stress markers HNE and HO-1 was performed using Axiovision image analysis software (Zeiss). Images of the entire hippocampus were prepared and all neurons of the CA1 and CA2 regions were analyzed and their relative densitometric values, minus the mean level of the surrounding neuropil, were determined. The mean neuronal staining levels for each mouse were calculated. The data was then decoded and the values for all the mice in the different genotype groups were averaged, and statistical significance of differences between the means was determined with one-way ANOVA or student's t-test.

## Results

**Figure 1:**
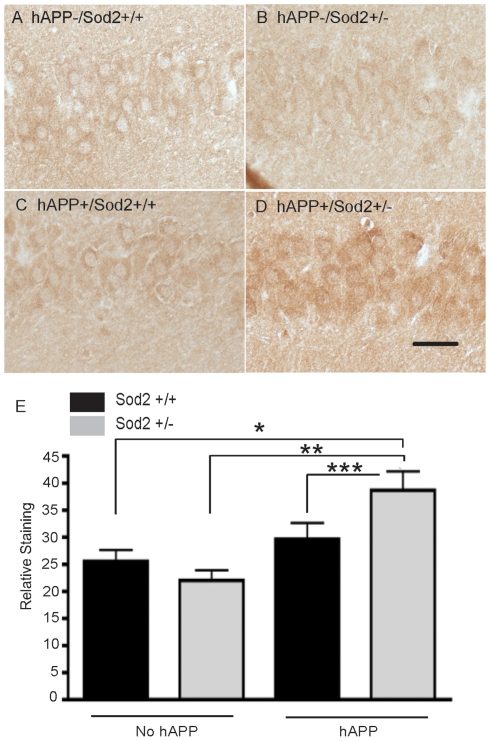
Sod2 reduction, in synergy with hAPP/Aβ expression, results in increased levels of the lipid peroxidation product HNE, in young (5–7 month-old) mice. Pyramidal neurons in the CA1 region of the hippocampus exhibit similar levels of HNE immunostaining in the hAPP-/Sod2^+/+^ (A), hAPP-/Sod2^+/−^ (B), and the hAPP+/Sod2^+/+^ (C) mice, whereas the hAPP+/Sod2^+/−^ mice exhibit increased levels of HNE (D). Quantification of the intensity of the neuronal HNE levels using densitometric analysis reveals the hAPP+/Sod2^+/−^ mice have significantly higher levels of neuronal HNE adducts as compared to hAPP+/Sod2^+/+^, as well as the hAPP-/Sod2^+/+^, hAPP-/Sod2^+/−^ mice (E). Bars represent mean + SEM, n = 5−7 per group *p<0.05, **p<0.01 by one-way ANOVA + Tukey Kramer post hoc analysis. ***p<0.05 by a one-tailed student's *t*-test.

In young mice (5–7 months), we compared the accumulation of the oxidative stress markers in the brain of mice representing the 4 different genotypes. Genotype-dependent differences in neuronal levels of the HNE adduct were readily apparent between the groups of young mice (Figure 1A–D). Specifically, quantification revealed neuronal densities of the HNE adduct were significantly increased in the hippocampus of hAPP transgenic mice with reduced Sod2 activity (hAPP/Sod2^+/−^), but not in hAPP transgenic mice with normal Sod2 activity (hAPP/Sod2^+/+^), or in Sod2^+/+^ and Sod2^+/−^ controls. However, HNE staining in mice without hAPP was unaffected by the ablation of one Sod2 allele (Figure 1E). Notably, neither hAPP expression alone (compare Sod2^+/+^ mice to hAPP/Sod2^+/+^ mice) nor Sod2 reduction alone (compare Sod2^+/+^ to Sod2^+/−^ mice) was sufficient to increase neuronal HNE accumulation in young mice.

**Figure 2:**
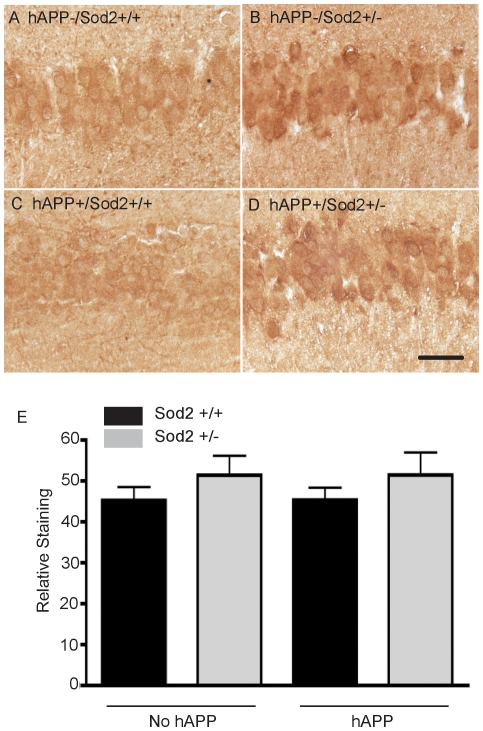
Neuronal HO-1 immunostaining in the pyramidal cells of the hippocampus of young (5–7 month-old) mice (A–D). Densitometric quantitation demonstrates that HO-1 levels tend to be higher in Sod2^+/−^ and hAPP/Sod2^+/−^ mice relative to mice with normal Sod2 levels (Sod^+/+^ and hAPP/Sod2^+/+^) (E). Bars represent the mean + SEM, n = 5−7 per group.

Upon qualitative assessment, levels of neuronal HO-1 in the young mice (5–7 months of age) appeared higher in hippocampal neurons of mice with ablation of one Sod2 allele, irrespective of hAPP expression (Figure 2A–D). Quantification, however, revealed only a slight increase in neuronal staining in these young mice with the Sod2^+/−^ mutation (Figure 2E) that did not reach significance.

**Figure 3:**
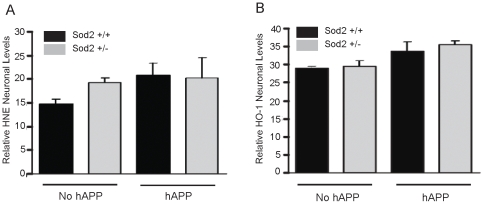
Immunostaining and densitometric quantification of HNE(A) and HO-1 (B) in pyramidal neurons in old (25–30 month-old) mice shows that these oxidative stress markers tend to increase slightly with APP/Aβ expression, although levels are not affected by reduction of Sod2 activity. Bars represent the mean + SEM, n = 5−7 per group.

Unexpectedly, the results obtained for the older mice (25–30 months of age) contrasted with the results from the younger mice. For example, neuronal HNE (Figure 3A) and HO-1 (Figure 3B) staining in old mice revealed no significant differences between the groups. Whereas the levels of both HNE and HO-1 tended to increase slightly with hAPP expression, there was no effect on levels of these oxidative damage markers with the ablation of one Sod2 allele at this age in the pyramidal neurons.

**Figure 4:**
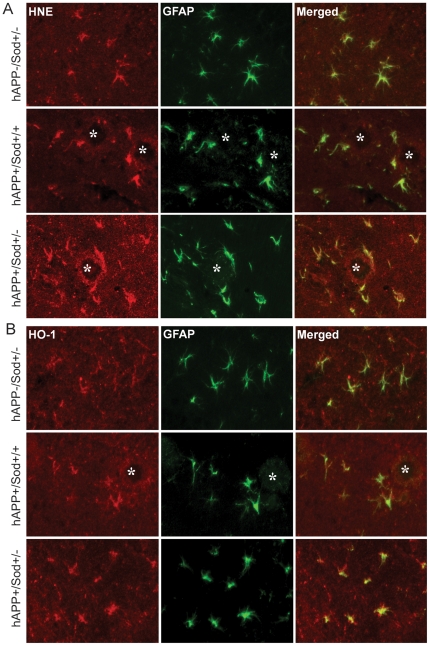
High magnification images of the double immunofluorescence staining in the hippocampus of Sod2^+/−^, hAPP/Sod2^+/+^, and hAPP/Sod2^+/−^ mice at 25–30 months of age using antibodies against GFAP (Green fluorescence), HNE (Red fluorescence in A), and HO-1 (Red fluorescence in B). In Sod2^+/−^ mice there is a moderate level of astrocytic (GFAP) staining, and moderate levels of HNE (A) or HO-1 (B) localized to glial cells (Merged images). In hAPP/Sod2^+/+^ and hAPP/Sod2^+/−^ mice there is extensive plaque formation (Asterisk), striking increases in levels of glial HNE(A), and HO-1 (B) relative to mice without hAPP/Aβ expression. The magnification is 20×.

Since previous studies using these mice documented changes in gliosis in old animals, we measured HNE and HO-1 levels in glial cells. In Sod2^+/+^ mice minimal astrocytosis was detected with an antibodies to glial fibrillary acidic protein (GFAP), HNE, and HO-1 (data not shown). In old mice that lack mutant hAPP expression there was moderate levels of astrocytosis in Sod2^+/−^. Consistent with previous results [15], the greater levels of astrocytosis in old hAPP transgenic mice was lessened by Sod2 reduction. These astrocytes also contained significant levels of HNE (Figure 4A) and HO-1 (Figure 4B). High magnification images of the double-label fluorescent microscopy confirmed that many of the cells with accumulated oxidative damage are in fact astrocytes containing GFAP. In young mice, astrocytosis was minimal as indicated by low GFAP expression and no significant Aβ deposition (data not shown).

## Discussion

Oxidative stress emanating from the mitochondria has been proposed to be a key pathogenic trigger in the progression of the neuronal deficits that characterize AD. Moreover, the activity of Sod2 has been reported to be reduced in AD brains [21]. In support of this hypothesis, previous studies in hAPP transgenic mice have shown that the reduction of Sod2 activity accelerates the onset of AD-related behavioral deficits, alters amyloid deposition, worsens the severity of synaptic density deficits and neuritic dystrophy, enhances microgliosis and increases the activity of the redox-sensitive transcription factor NFκB in the brains of these mice. Overt increase in oxidative damage resulting from the Sod2 reduction, however, was not initially detected in these mice using traditional biochemical methods, which suggests that the oxidative damage might be specific and/or focal. Furthermore, since aging hAPP transgenic mice undergo further oxidative changes and also exhibit a myriad of possible compensatory mechanisms that might mask the effects of the Sod2 mutation, we hypothesized that it is an earlier event of oxidative stress in the hAPP/Sod2^+/−^ mice that likely contributes to the pathology and behavioral changes seen in this model.

To address this question, we assessed the levels of two widely accepted and sensitive measures of oxidative damage, the lipid peroxidation product HNE and the enzyme HO-1, in the brains of both young (5–7 month old) and old mice (25–30 month old). We showed that in the hippocampus neuronal HNE levels were increased dramatically in young hAPP transgenic mice with reduced Sod2 activity. This result strongly suggests a possible synergistic effect of decreased Sod2 activity and mutant hAPP/Aβ expression, yielding significantly increased oxidative damage in neurons of young mice. Importantly, since no amyloid plaque deposition was found in young mice, this result indicates that the increase of oxidative stress at this age is Aβ plaque independent but dependent on Sod2 activity.

Chronic mitochondrial oxidative stress impairs mitochondrial function, including oxidative phosphorylation [14], that can, in turn, cause neuronal energy (ATP) deficits, negatively impact endogenous cellular repair systems [22], inhibit axonal transport [23], impair synaptic transmission [24], [25] and further increase reactive oxygen species production [26]. Mitochondrial dysfunction and oxidative stress occur early in all major neurodegenerative diseases, and there is strong evidence that this dysfunction has a causal role in pathogenesis [27]. Our study supports the conclusion that hAPP/Sod2^+/−^ mice are more susceptible at an early age to the accumulation of oxidative damage (HNE) which may result in their accelerated onset of AD-like phenotypes relative to hAPP mice with normal Sod2 activity [15].

Another oxidative stress marker, HO-1 induction, was subtly increased by the Sod2^+/−^ mutation, irrespective of hAPP/Aβ expression. The HO-1 immunoreactivity was intense in all animals, which may account for the inability to differentiate between hAPP- and hAPP+ pathology in different genotypes. Thus, in this study, HNE proved to be a more sensitive marker for oxidative damage in young hAPP/Sod2^+/−^ mice and suggests that lipid peroxidation is an acutely sensitive measure of oxidative damage in hAPP transgenic mice. These results are consistent with studies in humans in which lipid peroxidation products such as HNE, isoprostanes and neuroprostanes have proven to be reliable biomarkers of AD [28]–[30]. Overall, our current and previous data showing that the reduction of Sod2 activity in younger mice accelerated oxidative damage and hAPP/Aβ-related pathology supports the hypothesis that decreased Sod2 activity (and hence, increased O_2_^•−^) contributes to neuronal deterioration and impairment in AD.

In older mice in which Aβ deposition is apparent, Sod2 reduction did not significantly impact the oxidative damage that appears to be occurring as a result of hAPP/Aβ expression, implying that effect of Sod2 reduction on oxidative damage is saturated by a hAPP/Aβ-mediated pathogenic mechanism that occurs in the old mice. Alternately, it is interesting to note that an increase in Aβ deposition in AD patients brain is inversely correlated with neuronal oxidative damage measured by the level of 8-hydroxyguanosine, a major product of nucleic acid oxidation [31]. Therefore, although the mechanism is still unclear, it is tempting to propose that the accumulation of Aβ might reduce or hold steady the level of oxidative damage resulting from Sod2 reduction and consequently result in similar total levels of oxidative damage in both hAPP/Sod2^+/+^ and hAPP/Sod2^+/−^ brains. In fact, it is likely that other compensatory mechanisms in the brain may be upregulated with age and pathological progression in AD and other neurodegenerative diseases.

Another interesting and important finding is the increase of oxidative stress in the astrocytes of old mice. The oxidative stress accumulated within astrocytes is correlated with not only APP/Aβ but with Sod2 reduction, suggesting that the combination of Sod2 reduction and APP/Aβ expression augments oxidative stress in astrocytes although its mechanism and pathological implication remain to be elucidated. However, it might be possible that oxidative stress causes astrocytic damage, leading to impaired function including those functions that support neurons [32], and ultimately contributing to the development of AD pathology. In support of this notion, increased oxidative stress and DNA damage in astrocytes in AD has been reported [33], [34].

Our study provides strong support that neurons are the target of the oxidative insult caused by the partial reduction in the main mitochondrial superoxide scavenger Sod2, which accelerates the onset of mutant APP-dependent behavioral abnormalities in J20 mice [15] that are apparent even at 6–7 months of age [35]. Although the precise mechanisms by which Sod2 reduction promotes Aβ-induced neuronal deficits remains to be determined, our study suggests that reduction in the mitochondrial superoxide scavenger leads to the degeneration of neuronal integrity in the early stage of the disease. Supporting this hypothesis, the overexpression of Sod2 improved the cognitive functions and reduced Aβ accumulation in mutant hAPP transgenic mice [36]. Thus, retaining full activity of mitochondrial antioxidant defenses may be critical in preventing the early onset of AD symptoms whereas bolstering mitochondrial antioxidant potential and overall function may halt the progression of AD.
